# The Enhanced Recovery after Surgery (ERAS) Pathway Is a Safe Journey for Kidney Transplant Recipients during the “Extended Criteria Donor” Era

**DOI:** 10.3390/pathogens11101193

**Published:** 2022-10-16

**Authors:** Roberta Angelico, Francesca Romano, Camilla Riccetti, Marco Pellicciaro, Luca Toti, Evaldo Favi, Roberto Cacciola, Tommaso Maria Manzia, Giuseppe Tisone

**Affiliations:** 1 Transplant and HPB Unit, Department of Surgical Sciences, University of Rome Tor Vergata, 00133 Rome, Italy; 2 Kidney Transplantation, Fondazione IRCCS Ca’ Granda Ospedale Maggiore Policlinico, 20122 Milan, Italy; 3 Department of Clinical Sciences and Community Health, Università degli Studi di Milano, 20126 Milan, Italy; 4 King Salman Armed Forces Hospital, Tabuk 47512, Saudi Arabia

**Keywords:** enhanced recovery after surgery, kidney transplantation, complications, infections, recovery, hospital stay, allograft outcomes, patient outcomes

## Abstract

Enhanced recovery after surgery (ERAS) protocols are still underused in kidney transplantation (KT) due to recipients’ “frailty” and risk of postoperative complications. We aimed to evaluate the feasibility and safety of ERAS in KT during the “extended-criteria donor” era, and to identify the predictive factors of prolonged hospitalization. In 2010–2019, all patients receiving KT were included in ERAS program targeting a discharge home within 5 days of surgery. Recipient, transplant, and outcomes data were analyzed. Of 454 KT [male: 280, 63.9%; age: 57 (19–77) years], 212 (46.7%) recipients were discharged within the ERAS target (≤5 days), while 242 (53.3%) were discharged later. Patients within the ERAS target (≤5 days) had comparable recipient and transplant characteristics to those with longer hospital stays, and they had similar post-operative complications, readmission rates, and 5 year graft/patient survival. In the multivariate analysis, DGF (HR: 2.16, 95% CI: 1.08–4.34, *p* < 0.030) and in-hospital dialysis (HR: 3.68, 95% CI: 1.73–7.85, *p* < 0.001) were the only predictive factors for late discharge. The ERAS approach is feasible and safe in all KT candidates, and its failure is primarily related to the postoperative graft function, rather than the recipient’s clinical status. ERAS pathways, integrated with strict collaboration with local nephrologists, allow early discharge after KT, with clinical benefits.

## 1. Introduction

Enhanced recovery after surgery (ERAS) protocols have achieved a growing consensus in many different surgical specialties due to their benefits related to the significant reduction in length of hospital stay (LOS) and incurred costs, while maintaining low morbidity and increasing patient satisfaction [1,2]. ERAS protocols or “fast-track programs”, designed through a multidisciplinary approach, have now led to an enormous change in the perioperative care of many distinctive surgical operations [3,4], while they have been reluctantly adopted for patients affected by end-stage renal disease (ESRD) undergoing kidney transplantation (KT). 

KT recipients have traditionally been considered fragile individuals due to their multiple comorbidities, anesthesiologic risks, and complexity of postoperative care, including immunosuppression therapy and risk of delayed graft function (DGF) [5,6,7,8]. 

So far, there is no international consensus or any guideline on ERAS protocols in KT. Only a few single-center studies have reported on fast-track protocols and ERAS application in KT to date [8,9]. Preliminary results have suggested that the application of ERAS models in KT may have the benefits of shorter length of in-hospital stay and a low rate of postoperative complications, but no details on infective complications have been reported [2,9,10,11,12]. Furthermore, it is not yet clear how to identify KT candidates who are suitable for an ERAS approach. Moreover, in the current era, there is an increased use of grafts from of “extended criteria donors” (ECD) to face the organ shortage, which might prolong LOS due to the occurrence of DGF [13,14,15].

In the last decade, at our center, ERAS protocols have been constantly adopted in KT in the attempt to achieve early postoperative recovery, a low rate of postoperative infection, short hospital stays, and fast resumption of daily activities. The aims of the current study were to analyze the feasibility and clinical safety of ERAS protocols after KT during the ECD era in terms of postoperative complications, post-transplant infections, and readmission rates within 1 year of surgery, as well as to identify the risk factors of prolonged hospitalization within the ERAS program after KT.

## 2. Materials and Methods

### 2.1. Study Design

This was a single-center retrospective study that included all consecutive patients who underwent KT at the Transplant Center of the University of Rome Tor Vergata, Rome, Italy from January 2010 to December 2019, with at least 3 months of follow-up after transplantation. Patients whose follow-up was less than 3 months after KT, patients who received a simultaneous liver–kidney or pancreas–kidney transplantation, and those aged under 18 years at the time of KT were excluded from our study.

The study was approved by our local Ethical Committee and registered under research registry 37.21.

### 2.2. Data Collection

Recipient data at the time of transplantation [age, gender, body mass index (BMI), time on waiting list, causes of ESRD, and comorbidities], donor variables (type, age, cause of death, and comorbidities) and graft/transplant features [single or dual, re-transplantation, number of KT, sequential KT, pre-implant renal biopsy, and cold ischemia time (CIT)] were analyzed. 

ECD was defined as donor age ≥ 60 years or donor age ≥ 50 years with at least two of the following donor variables: arterial hypertension on chronic medical treatment, death from cerebrovascular cause, or pre-procurement creatinine serum level ≥1.5 mg/dL [16].

When performed, pre-implantation graft biopsy was assessed using the Italian necro-kidney score, which is based on the percentage of sclerosed glomeruli (grade: 0–3), tubular atrophy (grade: 0–3), interstitial fibrosis (grade: 0–3), and atherosclerosis (grade: 0–3), giving a total score from 0 to 12 [17]. According to the Italian national rules, pre-implantation kidney biopsy was always performed for donors aged over 65 years. During the initial study period (2010–2012), kidneys with a score of 3 or lower were used as single transplants, while those with a score of 4 and 5 were used as dual transplantations, on the assumption that the sum of the viable nephrons in the two kidneys approached the number of one ideal kidney. Since 2013, kidneys with a score of 4 were also allocated for single transplantations; grafts with a score of 5 were allocated as single or dual transplants depending on the predominant histological component of the score [18].

### 2.3. Surgical Technique 

According to the center’s practice, the transplanted kidney was placed in the right or left iliac fossa. After the preparation of the retroperitoneal fossa, the iliac arteries and veins were exposed, and the lymphatic vessels were ligated. The renal graft was anastomosed to the external or common iliac vessels. All ureterocystostomies were performed using the Lich–Gregoir technique with a double-J ureteral stent insertion [19]. In the case of double KT, ureters were anastomosed individually to the bladder. One perirenal drain was routinely positioned.

### 2.4. Postoperative Care and ERAS Protocol 

Before the ERAS implementation, no structured pathway existed in our transplant unit, and the absence of a standardized postoperative management determined a wide variability of LOS. Since January 2010, the ERAS program has been adopted in all patients undergoing KT. The ERAS protocol is composed of four phases including counseling on listing for KT, the preoperative phase, intraoperative phase, and postoperative period. The aim of our fast-track protocol is an early postoperative recovery and discharge home within 5 days of surgery, independently of graft function. The key steps of the ERAS protocol are summarized in Table 1.

### 2.5. Immunosuppressive Regimen

Depending on panel reactive antibody (PRA) score, the postoperative immunosuppressive regimen was based on induction with basiliximab (20 mg intraoperatively and on the fourth postoperative day) or antithymocyte globulin (three doses of 1.5 mg/kg for each dose) and maintenance therapy with tacrolimus once daily (0.15 mg/kg/day), mychophenolate mofetil (500–1500 mg/day) or sodium (360–1440 mg/day), and steroids (20 mg/day tapered to 5 mg/day within 3 months). A tacrolimus trough level of 7–9 ng/mL was aimed for within the first month after KT, along with 6–8 mg/mL within 6 months, and 5–6 ng/mL thereafter.

### 2.6. Outcomes 

Post-KT outcomes were evaluated in terms of duration of hospital stay, incidence of DGF, early (≤3 months) postoperative complications, readmission rate within 1 year from transplantation, and 5 year patient and graft survival. DGF was defined as the need for dialysis during the first week post KT or increased levels of creatinine above 2.5 mg/dL at postoperative day 10 [20,21]. Postoperative complications were classified as infectious (urinary tract infection, cytomegalovirus infection, or BK virus infection), vascular (renal artery stenosis, renal artery thrombosis, renal artery dissection, iliac artery dissection, renal vein thrombosis, iliac artery stenosis, pseudoaneurysm formation, or hematomas), or urological (ureteral stricture, urinary leak, vesicoureteral reflux, urolithiasis, bladder outlet obstruction or urinary tract obstruction from lymphocele); for each complication, the treatment chosen was recorded. Postoperative numbers of dialysis sessions during the hospital admission and after discharge, as well as the number of outpatient clinic reviews, were also recorded for each patient.

### 2.7. Statistical Analysis 

We retrospectively collected data in a consecutive database. All statistical tests were run using IBM SPSS 26.0 Software (IBM, Inc., Chicago, IL, USA) for Windows. Continuous variables were reported as medians with ranges or means ± standard deviation. Categorical variables were described as numbers and percentages. Normally distributed continuous data were analyzed using a parametric test (Student’s *t*-test). The Mann–Whitney U test and Fisher’s exact test were used for univariate analysis, and Cox multiple regression analysis was used for multivariate analysis. Patients were divided into two groups, according to the time of discharge after KT (early discharge group: discharge occurred ≤5 days after KT; late discharge group: discharge >5 days after KT), and comparisons between these two groups were performed in terms of baseline characteristics and outcomes. We used the Kaplan–Meier method to assess the influence of hospital stay length on patient and graft survival. Multivariate log-rank tests were used to explore the risk factors for late discharge (>5 days) in all the study population and in the subgroup of patients receiving ECD grafts. A *p*-value < 0.05 was considered significant.

## 3. Results

### 3.1. Study Population

Of the 500 KTs performed during the study period, 454 (90.8%) patients were included, while 46 (9.2%) patients were excluded because their post-transplant follow-up lasted <3 months (n = 39, 7.8%) or due to simultaneous liver or pancreas–kidney transplant (n = 7, 1.4%). The characteristics of recipients, donors, and transplants are detailed in Table 2.

A total of 290 (63.9%) patients were male, and the median age at the time of KT was 57 (19–77) years. The median BMI was 24 (15–37), and 50 (11%) recipients were obese, with a BMI ≥ 30. Glomerulonephritis, autosomal dominant polycystic kidney disease, and ESRD secondary to arterial hypertension were the most frequent indications for KT. At the time of transplantation, 203 (44.7%) recipients had at least one comorbidity, including arterial hypertension (n = 135, 29.7%), diabetes mellitus type 2 (n = 34, 7.5%), and cardiovascular diseases (n = 74, 16.3%). 

The majority (n = 440, 96.9%) of grafts were from deceased donors after brain death, while 14 (3.1%) were derived from living-related donors. The median donor age was 56 (11–88) years, and 225 (49.6%) were ECDs.

A single KT was performed in 434 (95.6%) cases, and 44 (9.7%) recipients received a second transplant. Before implantation, a graft biopsy was obtained in 225 (49.56%) cases, and, of those, 89 (39.6%) kidneys had a histological score >3. The median CIT was 11 (1–29) h, with 166 (36.6%) grafts having a CIT ≥10 h.

### 3.2. Outcomes

After transplantation, 174 (38.3%) recipients presented DGF. The median time of hospital stay was 6 (3–62) days, and 212 (46.7%) patients were discharged from the hospital within the ERAS target time (≤5 days). Of these, 87 (41.0%) KT recipients were sent home on the fourth postoperative day (POD).

After KT, in-hospital hemodialysis was required in 172 (38.3%) patients, while 46 (10.1%) patients underwent hemodialytic treatment at their own local center. For those who required post-transplant dialysis, the median number of dialytic sessions was three (1–9) during the hospital stay and two (1–12) at patients’ local center. During the first 3 months after hospital discharge, patients received a median of seven (0–16) clinical visits as outpatients.

A total of 122 (26.9%) KT recipients developed at least one postoperative complication within 3 months of KT, including 109 (24.0%) infections, 21 (4.5%) urological complications, and two (0.4%) vascular complications. In terms of infectious disease cases within 3 months of KT, 105 (23.1%) recipients presented cytomegalovirus (CMV) infection, 28 (6.2%) presented a urinary tract infection, and nine (2.0%) patients developed BK virus infection. The treatments of postoperative complications are detailed in Table 3.

Of the 156 (34.4%) patients readmitted to hospital within 1 year of KT, the majority (n = 122, 78.2%) were hospitalized within 3 months of KT, and the median time to readmission was 42 (1–352) days after transplantation. Among readmitted patients, 113 (72.4%) were re-hospitalized only once, 52 (33.3%) patients were re-hospitalized twice, and 32 (20.5%) patients were re-hospitalized three times or more. Causes of readmission to hospital are detailed in the Supplementary Materials (Table S1). 

The median follow-up time was 63 (3–140) months. At 5 years of follow-up, the median creatinine was 1.6 (0.66–14.9) mg/dL. Overall, the 5 year patient and graft survivals were 94.3% and 85.2%, respectively.

### 3.3. Risk Factors for Late Discharge within the ERAS Protocol 

In order to identify KT recipients who did not attain the ERAS discharge target (≤5 days post surgery), characteristics of patients sent home later than 5 days after KT (n = 242, 53.3%) were compared to those of recipients discharged within 5 days of transplantation (n = 212, 46.7%), as detailed in Table 4.

On univariate analysis, KT recipients with hospital stays longer than 5 days were older [58 (23–74) years vs. 54 (19–77) years, *p* = 0.003], had a greater BMI [25 (15–38) vs. 23 (16–37) years, *p* = 0.008], and had more comorbidities [126 (51.5%) patients vs. 77 (36.3%) patients, *p* < 0.001] compared to patients who could be discharged by POD 5. Patients who required a prolonged hospital stay received renal grafts from older donors [59 (15–83) vs. 53 (11–88%), *p* = 0.002], mainly from ECD [136 (56.2%) vs. 89 (42%), *p* = 0.003], and were more frequently exposed to CIT >10 h [100 (41.3%) vs. 66 (31.1%), *p =* 0.025].

**Figure 1:**
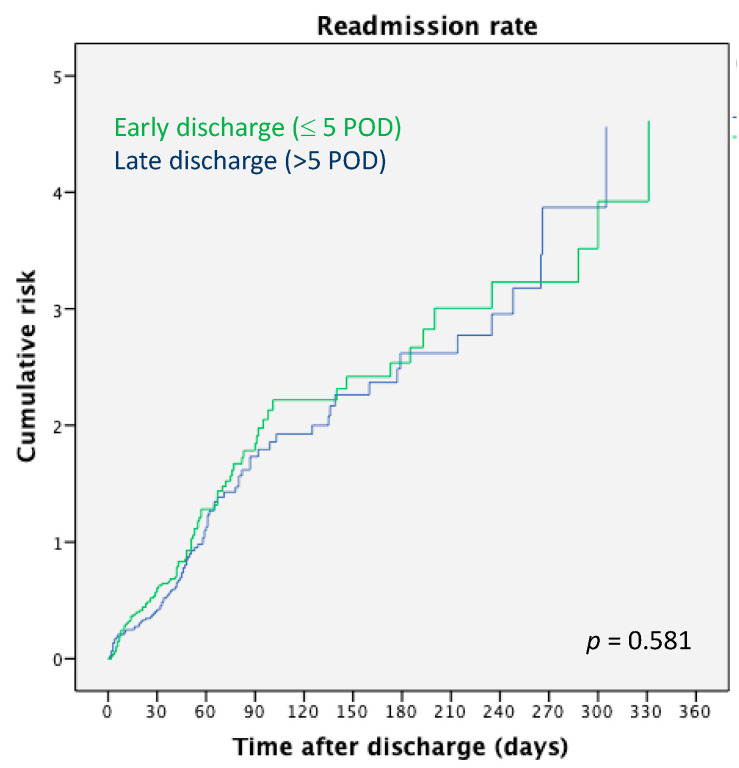
Readmission rate relying on time of discharge after kidney transplantation.

Patients who were discharged late after KT had a higher incidence of DGF compared to KT recipients with an early discharge [136 (56.2%) vs. 38 (17.9%), *p* < 0.0001] and more frequently required postoperative in-hospital hemodialysis [139 (57.4% vs. 35 (16.5%), *p* = 0.0001]. Thus, no differences were observed between the two groups in terms of early postoperative complications (infection, vascular, and urological), as well as readmission rate at 3 months and 12 months after transplantation. Patients with DGF had similar CIT when compared to these without DGF [11.5. (1.0–29.0) h vs. 10.6 (1.0–24.8) h, *p* = 0.337]. Among the readmitted patients, the time of readmission after KT was similar between the two groups (Figure 1). Moreover, after discharge, the median number of outpatient visits was similar for patients sent home within 5 POD and those sent home later [7 (0–19) vs. 7 (0–16), *p* = 0.822]. Graft function was also similar at 5 years of follow-up [1.52 (0.6–614.9) mg/dL vs. 1.70 (0.67–12.0) mg/dL, *p* = 0.473].

**Figure 2:**
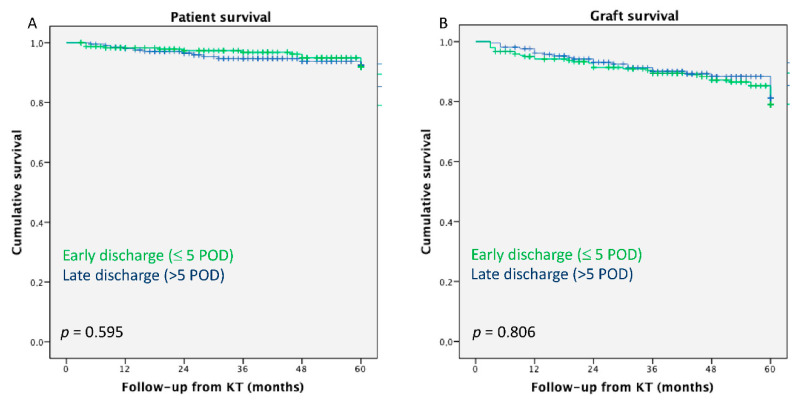
(**A**) The 5 year patient survival after kidney transplantation; (**B**) the 5 year graft survival after kidney transplantation.

KT recipients who were discharged early after KT showed comparable 5 year patient (94.2% vs. 94.3%, *p* = 0.806) and graft (87.3% vs. 83.5%, *p* = 0.595) survival to those who were discharged within the first 5 days after transplantation (Figure 2). 

On multivariate analysis, DGF (HR: 2.16, 95% CI 1.08–4.34, *p* < 0.030) and in-hospital dialytic treatment (HR: 3.68, 95% CI 1.73–7.85, *p* < 0.001) were identified as the only significant risk factors for late discharge after KT (Table 5).

### 3.4. Sub-Analysis of Patients Receiving Extended Donor Criteria Grafts

**Figure 3:**
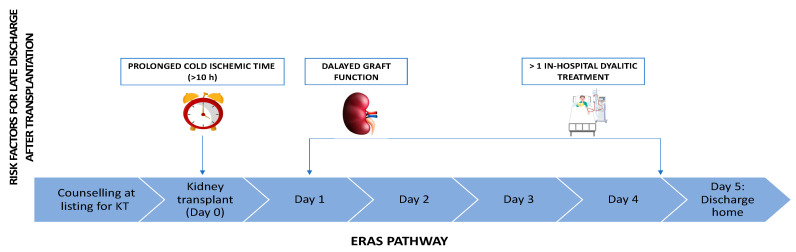
Risk factors for prolonged hospital stay after kidney transplantation in ERAS pathway.

Out of 225 KT recipients (49.6%) transplanted with ECD grafts, 136 (60.4%) patients could not be discharged within the ERAS criteria. At the univariate analysis, patients with prolonged hospitalization received graft with longer cold ischemia time [CIT > 10 h: 62 (45.6%) vs. 25 (28.1%), *p* = 0.012], experienced more DGF [79 (58.1%) vs. 19 (21.3%), *p* = 0.000] and required more in-hospital dialytic treatment [72 (58.1%) vs. 13 (14.6%), *p* = 0.000] when compared to patients discharged within ERAS criteria (Table S2). At the multivariate analysis, presence of the recipient’s arterial hypertension at the time of transplantation [HR: 3.02, 95% CI: 1.49–6.12, *p* = 0.002] and receiving in-hospital dialysis [HR: 3.70, CI 95%: 1.38–9.92, *p* = 0.009] were independent predicting factors of late discharge (Table S3). Risk factors for prolonged hospitalization after KT are summarized in Figure 3.

## 4. Discussion

In recent decades, several surgical subspecialties have incorporated the original concept postulated by Kehlet in 1997 that states that the “inclusion of multiple changes in perioperative practice could significantly improve outcomes” [22]. In this scenario, enhanced recovery pathways had a massive impact on perioperative care, leading to economic and social benefits without compromising (or even improving) clinical outcomes. A reduction in LOS, an improvement in quality of life and patient satisfaction, and a rapid return to daily activities are considered the driving forces of the growing consensus achieved by ERAS protocols [23,24,25].

Furthermore, the economic benefits associated with ERAS pathways have been estimated to be extremely positive when compared to “traditional” postoperative care. For example, in France, the hospital cost reduction was recently estimated at 1.8 million EUR for each percentage increase in ERAS activity, across several surgical fields [26]. 

However, ERAS implementation has been considered insidious in solid organ transplantation. In this field, the first reported applications of ERAS protocols involved early postoperative extubation after liver transplantation, which has been shown to be associated with a reduction in complication rates related to mechanical ventilation, such as pneumonia, and decreased costs due to a decline in intensive care unit requirements [27,28,29]. Notably, pre-transplant sarcopenia was generally identified as a predictive factor for ERAS failure [30]. 

In KT, the experience of ERAS protocols relies only on recent single-center series [8,9,10,11,12]. This limited expansion is due to several factors: firstly, ESRD patients are considered frail individuals, having many comorbidities [American Society of Anesthesiologists (ASA) grade III] and a high dialysis-related mortality rate (cardiovascular causes accounting for about 50% of deaths) [31]; secondly, KT recipients require immunosuppressive regimens after surgery, with the associated increased risk of infection and delay of wound healing [32,33]; lastly, KT recipients may develop DGF and need postoperative dialysis sessions [5].

In our center, we have consistently applied the ERAS approach to all KT recipients since 2010, with the aim of achieving rapid postoperative recovery and early discharge. Our protocol includes preoperative counseling of the KT candidate, followed by intraoperative and post-surgical management aimed at discharging the patient within 5 days of transplantation. In our experience, we could achieve this goal in 46.7% of patients, and almost half were discharged by POD 4.

With the present study, we demonstrated that the ERAS pathway could be implemented safely in all KT candidates. In fact, patients discharged within 5 days of KT had similar outcomes in terms of early postoperative complications and readmission rate compared to those discharged later. Moreover, 5 year patient and graft survivals were similar in recipients who were discharged within 5 days of KT and those discharged later. Moreover, previous reports demonstrated that the ERAS approach reduces LOS and readmission rate in both deceased- and live-donor graft KT recipients [2,8,9].

Regarding perioperative morbidity, we found no differences in terms of the number of infections within 3 months of KT across the early and late discharge groups. Risk factors of infectious complications after KT have been postulated in the literature, such as gender, age, concurrent comorbidities (including obesity and diabetes mellitus), long duration of catheterization, and immunosuppressive therapy [34]. It seems reasonable to predict that a shorter LOS could lead to a reduction in terms of exposure to nosocomial infections. However, an early discharge after KT does not exclude the need for close infection surveillance in the community of KT recipients through periodic clinical visits aimed at evaluating both clinical and laboratory findings. Strict postop monitoring will help in deciding on prompt hospital re-admission if there is a need for close monitoring, frequent blood work, samples for culture tests, or intravenous administration of medications. Conversely, during the follow-up of KT recipients, the prevention of CMV appears easier in the outpatient setting, with both universal prophylaxis and pre-emptive therapy strategies having been largely accepted [35]. Herein, we also observed that early discharge after KT is not associated with increased rates of urological complications, in agreement with Prionas et al., who showed that the implementation of enhanced recovery decreases urological complications, including ureteric stenosis, obstructions and urinary tract infections after KT when compared to control patients [36].

Our study demonstrated that the ERAS approach is feasible in all KT candidates, and its failure is primarily associated with the postoperative graft function, i.e., DGF and the number of post-transplant dialytic sessions, rather than the recipient’s clinical status. Although “frailty” has been postulated as a predictive factor for longer LOS in KT candidates [37], in our experience, we observed a higher rate of comorbidities in patients who needed a longer hospital stay after transplantation only in the univariate analysis, with this finding not being confirmed in the multivariate analysis. This suggests that, in the evaluation of ERAS viability in KT, the functional recovery of the renal graft should be integrated with the preoperative features of KT candidates. Moreover, in the subanalysis of patients transplanted with ECD grafts, the presence of recipients’ arterial hypertension at the time of transplantation was an independent predicting factor of failure of adherence to ERAS.

In the medical literature, patients who experienced DGF have traditionally been associated with longer LOS [38]. This approach can originate from a questionable belief that enhanced recovery programs are unsafe if DGF occurs, and that recipients who experience DGF cannot continue their follow-up in the community. Unfortunately, this further compounds the current scenario, where DGF is destined to unavoidably grow in the coming years due to the increasing use of marginal grafts from deceased donors [38,39]. Currently, the incidence of DGF after deceased-donor KT ranges from around 19% to 70% worldwide [13]. Several donor and surgical risk factors have been associated with DGF, including advanced donor age, higher kidney donor profile index, stroke/anoxia as donor cause of death, elevated donor serum creatinine, and prolonged cold ischemia [20,38,40]. Since it is well known that the occurrence of DGF leads to significantly higher costs over the course of the initial hospitalization [38], in clinical practice, this knowledge may potentially deter transplant centers from accepting grafts from ECD at risk of DGF. However, this is in contrast with the evidence that KT is cost-effective for healthcare systems when compared with long-term dialysis, regardless of the development of DGF [38,41]. Moreover, missing out on an organ offer in individuals suffering from ESRD, even a marginal graft, represents a disadvantage in terms of quality of life, morbidity and patient survival when compared to staying on dialysis replacement therapy [42]. In addition, in some countries, the choice to prolong the hospital stay of patients suffering from DGF after KT can also be associated with reimbursement policies relating to the hemodialysis program [12].

From our experience, we feel that KT recipients may be safely discharged soon after transplantation if they are clinically well, even when DGF occurs. In fact, in the present study, a sub-analysis limited to patients who developed DGF and were discharged within 5 days of transplantation showed similar outcomes compared to patients who had a longer hospital stay. Therefore, we believe that a broader use of higher-risk deceased donor kidneys should be encouraged in order to reduce organ discard and increase access to transplantation, alongside encouraging living donor kidney transplantation, which has been repeatedly associated with improved survival, economic benefits, and enhanced quality of life for patients with ESRD [41].

Clearly, the choice to prolong the hospital stay could be subsequent to the occurrence of postoperative complications managed through interventional radiology procedure or reoperation. However, a previous study of ERAS application in LT showed that perioperative care is associated more than surgery itself with better outcomes and shorter LOS [30]. This is evident also for KT, where several perioperative factors are considered predictive of keeping a patient in hospital following an uncomplicated transplantation, including parental analgesia, intravenous fluid for management of diuresis after transplant, intestinal dysfunction, parental immunosuppression, or inadequate immunosuppression medication threshold levels [9]. Additionally, the distance of the patient’s residence from the transplant center could preclude frequent clinic reviews as an outpatient, unless a local transplant clinic is available for their care [12]. To promote ERAS protocols, we strongly support close contact with a nephrologist from the patient’s own dialysis center, who could easily evaluate the patient after KT and define the correct management in agreement with the surgeon from the transplant center. Through this approach, we observed that patients undergoing early discharge had the same number of outpatient visits as those who were kept hospitalized for longer. In cases of post-discharge dialysis requirement, the return of the patient to their own local dialytic center is usually well perceived by patients and could potentially be cost-saving for the hospital healthcare system considering that the hospital stay’s costs are spared.

As already demonstrated in the setting of elective colorectal surgery [43,44], a multidisciplinary and multistep approach is essential for promoting successful ERAS programs in the setting of KT. In fact, several strategies described in the ERAS literature for other elective surgical procedures have been adopted in the solid organ transplantation field. The first principle is based on the patient’s compliance to the ERAS pathway. Although KT from deceased donors is an unplanned procedure, usually performed within a few hours of the admission to the hospital, during the pre-transplant assessment, clinicians should promote accurate patient counseling on ERAS pathways. A second tool consists of preoperative carbohydrate administration, which is known to reduce the postoperative catabolic phase, enhancing healing and potentially counteracting postoperative hyperkalemia in ESRD patients [8,45]. During the postoperative period, early mobilization, quick introduction of a solid diet, accurate pain control avoiding intravenous opioid administration, early bladder catheter removal (reducing the risk of infection in immunocompromised patients), and dedicated nursing care are key factors for ERAS success. Goal-directed fluid therapy represents another fundamental point of ERAS protocols in KT recipients, although it can be challenging, especially in anuric patients, given the pre-existing comorbidities [8]. In such patients, fluid balance should be conducted carefully, with daily measurement of body weight and arterial blood pressure, and physical examination of peripheral edema [12]. Central venous line insertion should be preferably avoided, as central venous pressure monitoring has been described as inaccurate and even inappropriate for the guidance of fluid therapy [46], and it could potentially represent an infection source. The use of transesophageal Doppler to monitor fluid balance in patients undergoing major operation has been recommended [47]. More recently, noninvasive monitoring of mean arterial pressure using the ClearSight™ system has been reported to be a good and easy option for intraoperative hemodynamic assessment [48].

Regarding the economic aspects, the costs of the postoperative stay together with the KT operation contribute to about one-third of the final yearly costs of transplantation; thus, any intervention that shortens the LOS after transplant will result in cost savings [9]. Therefore, it seems essential to optimize the outflow of the available economic sources of the healthcare system into standardized (although patient-tailored when necessary) programs of perioperative care.

This study is limited by its retrospective nature and does not include a control group of patients managed using a non-ERAS approach. However, since 2010, we have aimed to apply the ERAS pathway to all KT recipients at our center. Therefore, we chose not to compare patients with a historical cohort of KT recipients transplanted before 2010 because the two groups would be not similar with regard to many aspects, including immunosuppressive regimen and surgical technique. Despite our ERAS protocol defining that patients in good clinical conditions should be discharged from hospital independently from the graft function, we observed that, in clinical practice, this was not always applied. Another potential limitation is that we did not formally investigate the cost-effectiveness of the ERAS protocol, which could provide useful information. In fact, a detailed analysis should also evaluate the costs of all procedures, such as hemodialytic sessions or interventional radiology procedures, although ample variability among transplant centers is to be expected. Lastly, patients’ preoperative counseling on the ERAS pathway may represent a bias for the applicability of the program, but we believe that it is a crucial step to achieve an early recovery.

In the “era” of ECD grafts, whose use currently seems unavoidable given the organ shortage, our study shows that enhanced recovery protocols after surgery are feasible in all KT candidates and can be safely applied even in candidates with comorbidities and those receiving a marginal organ, without affecting early postoperative complications, infections, or readmission rates. In the medium term, patients discharged early after KT within the ERAS target time have 5 year graft and patient survivals similar to recipients discharged later. In a future perspective, further studies should investigate whether this strategy may potentially lead to cost savings, which could be more efficiently reinvested in the healthcare system.

## Figures and Tables

**Table 1:** ERAS protocol.

**Counseling at listing for KT**	Oral and written counseling Maintenance of weight and blood pressure
**Preoperative**	Oral and written counseling Additional dialysis session if last session completed >12 h ago
**Intraoperative**	Anti-embolism stockings Prophylactic antibiotics with IV cephalosporine Insertion of central line catheter only if needed Insertion of bladder catheter Insertion of one retroperitoneal drain Goal directed fluid therapy with the aim to obtain an MAP > 80 mmHg at the time of reperfusion Avoidance of vasoconstrictor agents if possible Early extubation (preferably on operative table) Short-acting anesthetic drugs Non-opioid analgesic regimen
**Postoperative**	Goal-directed IV fluid therapy (saline solution: 63 mL/h plus restoring of urine output for each hour) Accurate pain control (avoiding opioid analgesics and NSAIDs) Hemodialysis only for hyperkaliemia and fluid overload clinically relevant POD 1: − Doppler ultrasound of the renal graft − Oral feeds − Mobilization − Incentive spirometer − Calcium heparin (0.5 mg/kg/day) POD 2: − IV fluid suspension and removal of the central vein catheter (if present) − Start solid diet POD 3: − Bladder catheter removal − Retroperitoneal drain removal (if <100 mL/day output) POD 4–5: − Discharge home, independently from renal function − Education regarding drugs and urinating (output and frequency control) − Inform the nephrologist of the patient’s own dialysis center about outcome and schedule after KT Post discharge: − Outpatient clinic review with surgeons in 48 h with subsequent visits tailored to patient needs − Double J ureteral stent removal 6 weeks after KT by cystoscopy

Abbreviations: KT = kidney transplantation; MAP = mean arterial pressure; POD = postoperative day.

**Table 2:** Study population.

Variables	Number (%) or Median (Range)
Number of KT	454
**Recipient**	
Age (years)	57 (19–77)
Age (>60 years)	190 (41.9%)
Gender (male)	290 (63.9 %)
BMI	24 (15–37)
Obesity (BMI ≥ 30)	50 (11%)
Cause of ESRD:	
Glomerulonephritis	183 (40.3%)
ADPKD	96 (21.1%)
Arterial hypertension	48 (10.6%)
Other causes (unspecified, SLE, vasculitis, HUS, drug-induced nephropathy, cystinosis, oxalosis)	39 (8.6%)
Pyelonephritis	30 (6.6%)
Unknown ESRD	28 (6.2%)
Diabetes	19 (4.2%)
Congenital malformation	11 (2.4%)
Median time on waiting list (days)	675 (1–4760)
Comorbidities	203 (44.7%)
Arterial hypertension	135 (29.7%)
Cardiovascular diseases	74 (16.3%)
DMII	34 (7.5%)
Comorbidities ≥ 2	34 (7.5%)
**Donor**	
Type of donor:	
Donor after brain death	440 (96.9%)
Living-related donor	14 (3.1%)
Age (years)	56 (11–88)
Aged > 60 years	189 (41.6%)
Cause of death:	
Cerebral hemorrhage	285 (62.8%)
Head trauma	94 (20.7%)
Ischemic stroke	36 (7.9%)
Anoxic encephalopathy	27 (5.9%)
Others	12 (2.6%)
Comorbidities:	
Cardiovascular disease	79 (17.4%)
Arterial hypertension	175 (38.5%)
≥2 comorbidities	88 (19.4%)
Extended criteria donor	225 (49.6%)
**Transplant**	
Type of KT:	
Single KT	434 (95.6%)
Dual KT: (unilateral/bilateral)	20 (4.4%): 11 (2.4%)/9 (2%)
Re-transplant	44 (9.7%)
Sequential KT after LT	4 (0.9 %)
Pre-implant renal biopsy:	225 (49.56 %)
Renal biopsy score ≤3	136 (30%)
Renal biopsy score >3	89 (19.6%)
Median CIT (h)	11 (1–29)

Abbreviations: ADPKD = autosomal dominant polycystic kidney disease; BMI = body mass index; CIT = cold ischemia time; DMII = diabetes mellitus type II; ESRD = end-stage renal disease; HUS = hemolytic uremic syndrome; KT = kidney transplantation; SLE = systemic lupus erythematosus.

**Table 3:** Outcome and postoperative complications.

Outcomes	Number (%) or Median (Range)
Number of KT	454
Post-KT delayed graft function	174 (38.3%)
Median hospital stay (days)	6 (3–62)
Time of discharge:	
Early (≤5 days after KT)	212 (46.7%)
Late (>5 days after KT)	242 (53.3%)
Post-operative dialytic treatment:	
In-hospital	174 (38.3%)
At peripherical center	46 (10.1%)
Median number of dialytic treatments	0 (0–21)
1 year readmission rates after KT	156 (34.4%)
Early (≤3 months after KT)	122 (26.9%)
Late (4–12 months after KT)	34 (7.5%)
Median time of occurrence of readmission after KT (days)	42 (1–352)
Number of outpatient clinic visits within 3 months from KT	7 (0–16)
Early complications (≤3 months after KT):	122 (26.9%)
Infectious	109 (24.0%)
Urological	21 (4.5%)
Vascular	2 (0.4%)
Treatment of early complications:	
Surgical revision	16 (3.5%)
Graft explant	5 (1.1%)
Interventional radiology procedures:	39 (8.5%)
Rejection treatment	12 (2.6 %)
Percutaneous drainage of fluid (abscess, hematoma)	14 (3%)
Percutaneous nephrostomy and JJ ureteral stent placement	9 (1.9%)
Arterial stent placement	3 (0.6%)

Abbreviations: KT = kidney transplantation.

**Table 4:** Characteristics of patients with early or late discharge undergoing ERAS protocol.

Variables	Patients Discharged ≤5 Days after KT (n = 212)	Patients Discharged >5 Days after KT (n = 242)	*p*-Value
**Recipient**			
Age (years)	54 (19–77)	58 (23–74)	*0.003*
Age > 60 years	78 (36.8%)	112 (46.3%)	*0.045*
Gender (male)	135 (63.7%)	155 (64%)	1.000
BMI	23 (15–37)	25 (16–38)	*0.008*
Obesity (BMI ≥ 30)	21 (9.9%)	29 (12%)	0.549
Cause of ESRD:			0.052
Glomerulonephritis	101 (47.6%)	82 (33.9%)	
ADPKD	45 (21.2%)	51 (21.1%)	
Arterial hypertension	17 (8%)	31 (12.8%)	
Other causes (unspecified, SLE, vasculitis, HUS, drug-induced nephropathy, cystinosis, oxalosis)	15 (7.1%)	24 (9.9%)	
Pyelonephritis	15 (7.1%)	15 (6.2%)	
Unknown ESRD	10 (4.7%)	18 (7.4%)	
Diabetes	5 (2.4%)	14 (5.8%)	
Congenital malformation	4 (1.9%)	7 (2.9%)	
Median time on waiting list (days)	649 (1–4760)	700.5 (2–3821)	0.214
Comorbidities	77 (36.3%)	126 (51.1%)	*<0.001*
Arterial hypertension	49 (23.1%)	86 (35.5%)	
Cardiovascular diseases	26 (12.3%)	48 (19.8%)	
DMII	15 (7.1%)	19 (7.9%)	
Comorbidities ≥ 2	12 (5.7%)	22 (9.1%)	
**Donor**			
Type of donor:			1.000
Donor after brain death	(96.7%)	235 (97.1%)	
Living-related donor	7 (3.3%)	7 (2.9%)	
Age (years)	53 (11–88)	59 (15–83)	*0.002*
Age > 60 years	71(33.5%)	118 (48.8%)	*0.001*
Cause of death:			0.273
Cerebral hemorrhage	123 (58%)	162 (66.9%)	
Head trauma	48 (22.6%)	46 (19%)	
Ischemic stroke	20 (9.4%)	16 (6.6%)	
Anoxic encephalopathy	16 (7.5%)	11 (4.5%)	
Others	5 (2.4%)	7 (2.9%)	
Comorbidities:			
Cardiovascular disease	34 (16%)	45 (18.6%)	0.535
Arterial hypertension	75 (35.4%)	100 (41.3%)	0.210
≥2 comorbidities	41 (19.3%)	47 (19.4%)	1.000
Expanded criteria donor	89 (42.0%)	136 (56.2%)	*0.003*
**Transplant**			
Type of KT:			0.094
Single KT	(98.1%)	227 (93.8%)	
Dual KT (unilateral/bilateral)	4 (1.9%)	15 (6.2%)	
Retransplant	19 (9%)	25 (10.3%)	0.638
Sequential KT after LT	3 (1.4 %)	1 (0.4%)	0.344
Pre-implant renal biopsy:	87 (41.03%)	138 (57.02%)	0.001
Renal biopsy score ≤3	49 (56.3%)	87 (63%)	0.330
Renal biopsy score >3	38 (43.7%)	51 (37%)	
Median CIT (h)	10.2 (1.0–23.3)	11.5 (1.0–29.0)	0.068
CIT ≥ 10 h	66 (31.1%)	100 (41.3%)	*0.025*
**Outcomes**			
Post-KT delayed graft function	38 (17.9%)	136 (56.2%)	*<0.0001*
Median hospital stay (days)	5 (3–5)	8 (6-62)	*<0.0001*
Postoperative dialytic treatment:			
In-hospital	35 (16.5%)	139 (57.4%)	*0.0001*
At peripherical center	13 (6.1%)	33 (13.6%)	*0.008*
Early complications (≤3 months after KT)	55 (25.9%)	67 (27.7%)	0.750
Infectious	48 (22.6%)	61 (25.2%)	0.582
Urological	11 (5.2%)	10 (4.1%)	0.658
Vascular	1 (0.5%)	1 (0.4%)	1.000
1 year readmission rates after KT	67 (31.6%)	89 (36.8%)	0.276
Early (≤3 months after KT)	50 (23.6%)	72 (29.8%)	0.433
Late (4–12 months after KT)	17 (8.0%)	17 (7.0%)	
Median time of readmission after KT (days)	45 (1-238)	42 (2-352)	0.339
Number of outpatient clinic reviews within 3 months after KT	7 (1–15)	7 (0–16)	0.454

Abbreviations: ADPKD = autosomal dominant polycystic kidney disease; BMI = body mass index; CIT = cold ischemia time; DMII = diabetes mellitus type II; ESRD = end-stage renal disease; HUS = hemolytic uremic syndrome; KT = kidney transplantation; SLE = systemic lupus erythematosus. *p* values < 0.05 are expressed in italic format.

**Table 5:** Multivariate model evaluating predicting factors for late discharge after kidney transplantation.

Variables	HR	95%–CI	*p*-Value
Type of KT (single vs. dual)	3.04	0.91–10.11	0.072
CIT > 10 h	1.36	0.88–2.11	0.162
DGF	2.16	1.08–4.34	*0.030*
Recipient age	1.01	0.98–1.03	0.650
ECD	1.35	0.73–2.47	0.338
Donor age	0.99	0.98–1.0.23	0.944
In-hospital dialytic treatment	3.68	1.73–7.85	*0.001*

Abbreviations: CI = 95% confidence interval; CIT = cold ischemia time; DGF = delayed graft function; ECD = extended criteria donor; KT = kidney transplantation; HR = hazard ratio. *p* values < 0.05 are expressed in italic format.

## Data Availability

Data are available on request due to restrictions (privacy or ethical).

## Supplementary Materials

The following supporting information can be downloaded at https://www.mdpi.com/article/10.3390/pathogens11101193/s1: Table S1. Causes of readmission; Table S2. Univariate analysis of subgroup of KT recipients transplanted with extended criteria donor grafts; Table S3. Multivariate model evaluating predicting factors for late discharge after kidney transplantation in patients receiving ECD grafts.
