# Supplementary material for: The Enhanced Recovery after Surgery (ERAS) Pathway Is a Safe Journey for Kidney Transplant Recipients during the “Extended Criteria Donor” Era
Source: Pathogens. 2022 Oct 16;11(10):1193. doi: 10.3390/pathogens11101193 (PMC9610733; doi:10.3390/pathogens11101193)
Supplement: Supplementary file 1 [file pathogens-11-01193-s001.zip › pathogens-1895335-Supplementary.pdf]

Table S1: causes of re-admission.

Causes of re-admission	Early re-admission (<3 months after KT)	Late re-admission (4-12 months after KT)
Infections	49 (10.8%)	14 (3.1%)
ARF and electrolyte imbalances	23 (5.1%)	5 (1.1%)
Surgical complications	11 (2.4%)	1 (0.2%)
Cardiovascular complications	15 (3.3%)	3 (0.7%)
Cancer	1 (0.2%)	1 (0.2%)
Urological complications	12 (2.6%)	3 (0.7%)
Rejection	1 (0.2%)	1 (0.2%)
Other complications	10 (2.2%)	6 (1.3%)

Abbreviations: ARF = Acute Renal Failure.

Table S2: Univariate analysis of subgroup of KT recipients transplanted with extended criteria donor grafts.

Variables	Patients discharged ≤5 days after KT (n= 89)	Patients discharged >5 days after KT (n=136)	*p* value
**Recipient**			
Age (years)	63 (36-77)	63 (29-74)	0.396
Age > 60 years	62 (69.7%)	101 (74.3%)	0.451
Gender (male)	58 (65.2%)	87 (64.4%)	0.887
BMI	24 (17.7-32.6)	24.7 (16.8-33.3)	0.095
Obesity (BMI ≥30)	7 (7.9%)	12 (8.8%)	1.000
Cause of ESRD:			
• Glomerulonephritis	41 (46.1%)	47 (34.6%)	
• ADPKD	23 (25.8%)	26 (19.1%)	
• Arterial hypertension	6 (6.7%)	21 (15.4%)	
• Other causes (unspecified, SLE, vasculitis, HUS, drug-induced nephropathy, cystinosis, oxalosis)	4 (4.5%)	13 (9.6%)	0.144
• Pyelonephritis	6 (6.7%)	9 (6.6%)	
• Unknown ESRD	6 (6.7%)	8 (5.9%)	
• Diabetes	3 (3.4%)	10 (7.4%)	
• Congenital malformation	0 (0%)	2 (1.5%)	

Median time on waiting list (days)	623 (1-3773)	629 (2-3185)	0.957
Comorbidities			
• Arterial hypertension	16 (18%)	50 (36.8%)	*0.003*
• Cardiovascular diseases	12 (13.5%)	33 (24.3%)	0.061
• DMII	6 (6.8%)	14 (10.3%)	0.474
• Comorbidities ≥ 2	4 (4.5%)	14 (10.3%)	0.138
**Donor**			
Age (years)	65 (48-88)	66 (51-83)	0.594
Age > 60 years	67 (75.3%)	111 (81.6%)	0.314
Cause of death:			
• Cerebral haemorrhage	71 (79.8%)	108 (79.4%)	
• Head trauma	6 (6.7%)	14 (10.3%)	
• Ischemic stroke	9 (10.1%)	9 (6.6%)	0.660
• Anoxic encephalopathy	3 (3.4%)	5 (3.7%)	
• Others	0 (0%)	0 (0%)	
Comorbidities:			
• Cardiovascular disease	23 (25.8%)	40 (29.4%)	0.649
• Arterial hypertension	63 (70.8%)	80 (58.8%)	0.089
• ≥ 2 comorbidities	35 (39.3%)	40 (29.4%)	0.148
**Transplant**			
Type of KT:			
• Single KT	85 (95.5%)	122 (89.7%)	
• Dual KT (unilateral/bilateral)	4 (4.5%)	14 (10.3%)	
Re-transplant	8 (9%)	10 (7.4%)	0.802
Sequential KT after LT	2 (2.2%)	1 (0.7%)	0.564
Pre-implant renal biopsy:			
• Renal biopsy score ≤ 3	33 (47.8%)	36 (52.2%)	0.165
• Renal biopsy score > 3	66 (59.5%)	45 (40.5%)	
Median CIT (hours)	605 (285-1400)	732 (64-1380)	*0.026*
CIT ≥ 10 h	25 (28.1%)	62 (45.6%)	*0.012*
**Outcomes**			
Post-KT delayed graft function	19 (21.3%)	79 (58.1%)	*<0.001*

Median hospital stay (days)	5 (3-5)	7.5 (6-39)	*<0.001*
Post-operative dialytic treatment:			
• In-hospital	13 (14.6%)	72 (58.1%)	*<0.001*
• At peripheral centre	9 (10.1%)	15 (11%)	1.000
Early complications (≤ 3 months after KT)			
• Infectious	20 (22.5%)	41 (30.1%)	0.223
• Urological	10 (11.2%)	10 (7.4%)	0.345
• Vascular	1 (1.1%)	1 (0.4%)	0.396
1-year Re-admission rates after KT			
• Early (≤ 3 months after KT)	37 (41.6%)	54 (39.7%)	0.783
• Late (4-12 months after KT)	47 (52.8%)	63 (46.3%)	0.413
Median time of re-admission after KT (days)	48 (1-305)	43 (3-352)	0.299
Number of outpatient clinic reviews within 3 months after KT	7 (0-19)	7 (0-15)	0.899

Abbreviations: ADPKD= autosomal dominant polycystic kidney disease; BMI= body mass index; CIT=Cold ischemia time; DMII= Diabetes Mellitus type II; ESRD= End-stage renal disease; HUS= hemolytic uremic syndrome; KT= Kidney Transplantation; SLE= systemic lupus erythematosus.

Table S3: Multivariate model evaluating predicting factors for late discharge after kidney transplantation in patients receiving ECD grafts.

**Variables**	**HR**	**95%–CI**	**p value**
CIT ≥ 10 h	1.83	0.966–3.45	0.064
DGF	1.62	0.63–4.20	0.320
In-hospital dialytic treatment	4.78	1.69–13.47	0.003
Recipient's arterial hypertension	3.02	1.49–6.11	0.002

Abbreviations : CIT =Cold ischemia time ; DGF= delayed graft function
